# P-1757. Standardizing the Penicillin Allergy Documentation Program and Optimizing the Use of Beta Lactam Antibiotics In a Tertiary Care Hospital

**DOI:** 10.1093/ofid/ofae631.1920

**Published:** 2025-01-29

**Authors:** Linta Susan Kuriakose

**Affiliations:** Mary Queen's Mission Hospital, Kollam, Kerala, India

## Abstract

**Background:**

The adoption of standardized penicillin allergy assessment protocols and the optimization of beta-lactam antibiotic usage have been advocated by international guidelines on Antibiotic Stewardship Programs (ASPs). However, the precise effects of conducting these assessments on allergy documentation and antibiotic prescription practices remain inadequately characterized.

**Methods:**

A retrospective quasi-experimental study was devised to assess the repercussions of implementing a standardized penicillin allergy assessment initiative led by the Antimicrobial Stewardship Team, comprising Infectious Disease (ID) Physicians, clinical pharmacists, and nurses. Commencing January 2022, nursing personnel conducted comprehensive evaluations of hospitalized patients with documented penicillin allergies, with subsequent review by clinical pharmacists. The pre-intervention phase encompassed randomly selected adult patients with reported penicillin allergies admitted between January 2019 and December 2021. The principal study endpoint was the accurate delineation of penicillin allergies in the electronic health record (EHR), encompassing clarification of allergic reactions and removal of erroneously labelled allergies. Secondary endpoint was beta-lactam antibiotic utilization within 90 days of hospitalization.

**Results:**

A total of 360 patients were enrolled, comprising 140 during the intervention phase and 220 during the pre-intervention phase. The proportion of patients with updated allergy information surged from 22% to 53% post-implementation of the program (P < 0.0001), with inappropriate allergy designations rectified in 18 (5%) patients. Notably, the program's implementation did not alter the proportion of patients receiving beta-lactam antibiotics (22% vs. 25%; P = 0.51).

**Conclusion:**

The adoption of a standardized penicillin allergy assessment initiative exerted a significant influence on allergy documentation within the EHR, without imposing excessive burdens on clinical team. This initiative facilitated the updating of allergy information for the majority of patients and rectified inaccurately labelled penicillin allergies.

**Disclosures:**

**All Authors**: No reported disclosures

